# Patterns of Engagement and Non-Engagement of Young Fathers in Early Intervention and Safeguarding Work

**DOI:** 10.1017/S1474746415000573

**Published:** 2016-01

**Authors:** Harry Ferguson

**Affiliations:** School of Sociology and Social Policy, University of Nottingham E-mail: harry.ferguson@nottingham.ac.uk

**Keywords:** Early intervention, child protection, safeguarding, fathers, parenting, social care, Family Nurse Partnership

## Abstract

This article is based on research into early intervention and safeguarding work with young fathers. It draws on a study of the Family Nurse Partnership (FNP), a home visitation service in the UK that is offered to vulnerable teenage mothers. The research investigated whether and how such early intervention work was done with the fathers of these babies. Three broad patterns of engagement emerged from the research: (1) where fathers were fully engaged with the service straightaway and the relationship with the family nurse deepened over time; (2) where fathers were partially engaged with the service; and (3) where fathers were resentful at the outset and never stopped being resistant and sometimes hostile towards intervention. Within these broad patterns several nuanced aspects of professional-father relationships are identified, which are the product of the interaction of several factors. Some general implications for early intervention and safeguarding work with fathers and their babies are drawn out.

## Introduction

Although the literature on fatherhood, social policy and practice interventions has grown in recent years, it remains a commonplace finding that health and social care professionals tend to ignore men, who are often reluctant to engage with services (Featherstone *et al.*, 2007; Featherstone, 2009; Walters, 2011; Maxwell *et al.*, 2012). Typically this is said to be due to the nature of masculinity and how men regard it as a sign of weakness to seek or receive help (O’Brien *et al.*, 2005). Or the problem is said to lie with how welfare services operate on gendered assumptions of mothers as natural and primary caregivers, and often ignore men and their capacities to nurture (Scourfield, 2003; Brown *et al.*, 2009; Panter-Brick *et al.*, 2014). Some research is beginning to reveal a more complex picture of younger men's fathering that shows that most of those studied were very involved in their child's life, providing support and care to their partner during pregnancy and in early parenthood. But although some such men reported positive experiences, they often felt excluded or judged when accessing health and social care services (Ross *et al.*, 2010). Studies of child protection work in general (Ferguson, 2011), and high risk cases where babies and older children died, despite being known by professionals to be at high risk, have found that it was mothers who are worked with, while fathers tended to be ignored or allowed to opt out of interventions (Brandon *et al.*, 2008; Ofsted, 2011). Young fathers then may experience a double negative exclusion: first, because of the negative narrative about young men, particularly those with vulnerabilities on the margins of society who become young fathers; and second, because of the general exclusion of fathers (Quinton *et al.*, 2002). The combination may heighten the judgemental attitudes of practitioners and make young fathers particularly wary of services. The experiences of exclusion for young black fathers can be even more pronounced (Pollock *et al.*, 2005). The tendency is for policy and practice analyses to create simple stereotypes of the bad professional who fails to engage with the good father, or its opposite, of the good professional/bad father, where motivated workers try to engage men who are ‘hard to reach’. Research into professional–father relationships needs to do justice to the variety and complexity of these encounters and relationships and examine the dynamics of how social attitudes, policy assumptions, professional systems, practitioner outlooks, and service users contribute to the meanings and outcomes of interventions (Williams and Popay, 1999).

This article is based on research into early intervention and safeguarding work with young fathers and specifically the work of the Family Nurse Partnership (FNP). I will argue that such early intervention work points to the need for greater recognition of the complexity of engagement and non-engagement of fathers in social care and health work. The FNP is an early intervention service that ‘is offered to first time vulnerable teenage mothers’ (Department of Health, 2009: 8) and delivered by specially trained ‘family nurses’ who are qualified nurses from areas such as health visiting, midwifery and mental health. Delivered through home visits, the programme begins as early as possible in pregnancy and continues until the child's second birthday. The FNP is a licensed programme which was introduced in the UK in the 2000s, having been developed in the United States in the 1980s (Olds *et al.*, 1997, 2002). While there is a clinical element to family nurses’ work in how they advise on child and maternal health, their approach is social and therapeutic. They are trained in ‘motivational interviewing’ (Miller and Rollnick, 2002), which means that rather than imposing solutions by telling parents what they should do, they seek to enable them to make informed choices about child rearing and achieve change through in-depth engagement. A great deal of emphasis then is placed on the *relationship* between the practitioner and service user as a vehicle for change (Ferguson and Gates, 2015).

The FNP is a hugely significant policy initiative as it is the largest evidence-based family intervention programme in the UK. An earlier article outlined the broad research findings in terms of what the FNP does with fathers (Ferguson and Gates, 2015). The focus of this article is on three patterns of engagement/non-engagement that emerged from the research. Firstly, where fathers were fully engaged with the service straightaway and the relationship with the family nurse grew and deepened over time. Second, where fathers were partially engaged with the service; and finally where fathers were resentful at the outset and never stopped being resistant and sometimes hostile towards intervention. Within these broad patterns, several nuanced aspects to engagement will be identified. It will be argued that engagement, or the lack of it, must be understood as a process that is the product of the interaction of several factors. The research findings will be presented thematically and in case study form to enable crucial biographical information about fathers and the dynamics of their interactions with mothers, babies and professionals to be considered. As well as focusing on the specificities of the FNP intervention, the article will draw out some more general learning for early intervention and safeguading work with fathers and their babies.

## The study

The aim of the research was to evaluate whether and how the FNP worked with fathers and the men's experiences of the service. The FNP commissioned the research due to an awareness of the growing recognition of the importance of fathers in children's development (Lamb, 1997; Fatherhood Institute, 2013) and their uncertainty about how they engaged with fathers, and with the intention of learning about how they can increase men's involvement in their work. A mix of quantitative and qualitative research methods were used. The research took place in one FNP team, where there were seven family nurses and a supervisor. Each family nurse had a maximum caseload of twenty-five families who were visited on a weekly or bi-weekly basis, depending on the stage of the two-year programme. Family nurses were interviewed twice about their experiences of working with fathers and families to create a profile of each of their cases. Data about the fathers, their levels of involvement with their children and experience of the family nurses’ practice was gathered directly from fathers through a self-completion questionnaire. This was distributed by the nurses and returned in sealed envelopes so the men could be assured the nurse would not see their answers.

The FNP team were found to be actively involved with a total of 144 mothers/families. Thirty of these cases were discounted from the study because the father's identity or whereabouts was unknown, or because the fathers were known but it wasn't appropriate to contact them, most commonly due to domestic abuse. This left a survey sample of 114 fathers and fifty-four questionnaires were returned, a 47 per cent return rate. Interviews were then conducted with twenty-four fathers about their experiences of pregnancy, fatherhood and FNP intervention. The interview sample was purposively designed to reflect the diversity of men receiving the service. Four interviewed fathers were from BME backgrounds, while seven had social care involvement, in three of which the child was subject to a child protection plan. The fathers were also selected on the basis of what was known about their levels of engagement with the service. The survey and interview data showed that fathers’ levels of involvement with the service varied hugely, from very engaged to no engagement. The data are naturally biased towards fathers who were more engaged with the service, who were easier to access. Fifteen of the twenty-four interviewed fathers were heavily involved with the FNP. Intense efforts were made to reach fathers who had low levels of FNP engagement or none at all and nine such fathers were eventually interviewed. The interviews were semi-structured, lasted between sixty and ninety minutes, and most took place at the man's home; two were in prison.

## Negotiating the new identity of fatherhood

The survey findings show that men on FNP caseloads ranged in age from seventeen to thirty-four years. Almost a third (29 per cent) were teenage fathers, while some 58 per cent were aged twenty-one or under. The mothers ranged in age from sixteen to twenty-one, which is to be expected given that the service was targeted at first-time teenage mothers. The majority of the children (80 per cent) were one year old or younger and over a quarter (28 per cent) were under six months. Most of the men were the biological father of the child (85 per cent). For just over half (57 per cent) of the fathers the pregnancy was unplanned. The majority of men had low educational attainment, were unemployed and living in poverty because they had very low incomes or no income at all due to their young age. Many had either been in trouble with the youth or criminal justice system or been on the edge of it. Over a third had experienced parental separation as children (37 per cent), 70 per cent of which occurred before the men were ten years old. Eleven per cent of the fathers had been in care, and one was still in foster care at the time of the study.

This then was a cohort of young men who were highly vulnerable and who could easily be regarded as having limited potential to be responsible, caring fathers. Some fathers who avoided the researchers and the FNP seemed to conform to this stereotype, as they were regarded as a danger to their partners and babies. However, a powerful common aspect of all the narratives of the men in the study was that fatherhood and falling in love with their babies had changed them and given meaning and purpose to their lives.

I want to be there to buy him stuff. That is why I am doing a course so I can get a job. I wasn't really bothered, but as soon as she got pregnant I decided I needed to sort myself out. (Father 18; child 15 months)

This included a minority of men who had not been at the birth or ever engaged with the FNP because they were in prison. ‘Dave’ was interviewed in prison, where he was serving a nine-year sentence for serious violence against a member of the public. The couple only knew that Elaine was pregnant one week before he went to jail. He is visited weekly in prison by Elaine and the baby, and described a very unhappy childhood, which included being abused by his mother and prior to prison ‘living all over the place’. But he now feels fatherhood offers him a different life:

Being a father has definitely changed me. When I was in my last prison I used to fight every day. But since [my son] was born I’ve stopped. First thing that comes into my mind is my son. I’ve only had one fight here in the six months I’ve been here – a guy hit me in the mouth with a snooker ball and me and my mates battered him. Then they split us up.

I’m proud of being a dad. When I see [my child] and he smiles at me, it hurts me so much and when I hold him, it hurts me so much. I love him so much. I’ve got pictures of him all over my pad, I think about him every night. I speak to him on the phone every day. He laughs and goes ‘goo go gaga’.

Other research into imprisoned or recently released fathers shows how important acquiring a fatherhood identity and having a ‘generative’ desire to care for the next generation is for them (Erickson, 1963; Walker, 2010). However, their absence from birth and their child means that engaging in fathering is experienced largely at the level of desire. For all of the young men in this study, the critical issue was how this aspiration was turned into day-to-day fathering with their babies, or not, and the role that early intervention could play in helping them to do so.

## Fully engaged fathers

A feature of the stories of men who were living with, or in close daily contact with, their babies and fully engaged with the FNP programme was how becoming a father had not only changed their identity but their daily lives and practices. A striking finding was how effectively many of the vulnerable young men in the study had made (and were still making) the transition to fatherhood. The men who became actively involved in parenting were helped by early intervention to channel their commitment and love and gain a clear view of their role as fathers. This is typified by Keith, who was seventeen at the time of the research interview and father to nine month old James. Keith's girlfriend Mary (twenty) became pregnant two months after they started going out; it was unplanned. Keith officially lives with his mother, while Mary and James live in a rented house. They have plans to live together when they can afford to. Keith has no income whatsoever, no benefits because of his age, and relies entirely on hand outs from his mother. Mary's income (from benefits) supports their child. Like all of the men in the study who lived in poverty he felt deep guilt and sadness at not being able to buy even basic things for his child. He has no formal qualifications and has never worked. He had problems with anger and being verbally abusive at school (never physically, he says), and went on an anger management course which helped him greatly. He dropped out of school at fourteen, officially left at sixteen and very soon after learned that he was to become a father. He was scared, expecting fatherhood to be ‘really hard’. It has turned out to be easier, largely because he loves it.

Keith is at Mary's every day and spends several nights a week there. He said he gets involved in all aspects of parenting, apart from changing soiled nappies, which make him retch. This does not mean he has never had his son on his own; he had him for a whole weekend once and just had to persevere with the nappy changing. His son is ‘my life’. He gets huge pleasure from fatherhood and even from simply looking at his son for hours. Before becoming a father he spent much of his time out with his mates, and playing football. He has calmed and settled down now. He found it very hard at first and gave in to the feelings of wanting to be out and ‘free’. But he feels he has learned to be responsible and what his priorities are. Keith was very positive about the family nurse and how she has helped him. He is always there when she visits and feels that she gives him the same amount of time and attention as Mary. During the pregnancy, the nurse helped him to develop a better routine, not to stay up so late and to be more organised. He valued the books the family nurse gave him to read during the pregnancy as he knew nothing about babies or parenting. He trusts the family nurse and put great emphasis on how much she has helped him to understand his partner (who has had some post-natal depression), deal with his own feelings, helped them as a couple to communicate and work things out in their relationship and about how best to parent their son. He has learned a huge amount about practical child care, having initially been frightened to even hold the baby who was so small and vulnerable. His own mother is very helpful with the baby, and he is much closer to his father now than he was in the past. He intends to have more active involvement with his son than his father did with him, to ‘be there for him’.

Keith's experience typifies how active fathers regarded their motivation to become involved with their children as having increased significantly during the pregnancy and birth (Bunting and McCauley, 2004). Becoming a parent involves making a developmental transition and practicing a form of ‘univocal reciprocity’, which is a ‘type of moral norm that encourages individuals to engage in social exchanges with others without expecting to receive direct or immediate reciprocation’ (Marsiglio, 1995: 3). In engaging fathers from the time of the pregnancy, the FNP capitalised on this emotional energy and ethical commitment by helping the man to channel it into active skilled care for his baby. Many men spoke of how unprepared they felt for the role of fatherhood and how early intervention helped them to gain knowledge, skills and confidence:

Just looking after [child] in general, really. Knowing when she's ill, knowing the sad face, the happy face, the bored face, stuff like that really, it's been good . . . She's told us that obviously they don't like, they can tell when you’re angry by your voice, by your facial impressions, and she can tell when you’re like trying to communicate happy with her by your facial impressions and the sound of your voice if you know what I mean . . . she also let us know what sound is for what, I can't explain it, like sounds for the feelings, she's feeling, does that make sense? . . . sounds, like she would make a different kind of sound in how she's feeling, obviously besides the crying. When she's happy she makes like a high pitched voice, and chuckles to herself and that. (18 yr old; child 7 months)

She has helped when playing with and talking to the baby, showing us games and activities, like showing us how to encourage him to walk . . . She has helped me understand that [partner] needs a break, and how to avoid post-natal depression. (20 year old; child 11 months)

This shows how in addition to support with parenting, young fathers, new to pregnancy and also to long-term committed relationships with women, and certainly pregnant women, welcomed professional help with improving their knowledge and understanding of their partner's experience and needs, pre- and post-birth. Research bears out that women who have a positive relationship with their partners are less likely to suffer from post-natal depression. Kyle, the last quoted twenty-year-old White British father, spoke highly of the family nurse: ‘she makes sure we’re ok’ and rated her as ‘really important, the main thing’. While feeling helped with developing some parenting skills, Kyle regarded himself as still having more to learn ‘about the washing and clothing of the baby, as at the moment [partner] does all of that, because if I wanted to take him away for a few days, I’d need to know that’. While these men were, by family nurses’ accounts as well as their own, very involved in providing care for their babies, their narratives reveal the gendered nature of parenting. Kyle suggests he could not yet care for the baby without relying on what his partner does, while Keith's partner Mary is the resident primary parent, and his aversion to soiled nappies implies that this mother has little or no no choice about doing all such tasks.

These data also show how assessments of fathers’ levels of involvement with their children and the status of being an ‘engaged father’ with professional services are contested. The family nurse's perspective was that Kyle was not engaging well with her or the programme. Yet according to Kyle, when the family nurse visited he stayed in the room, felt included and learned a lot. But he was less reliant on her for help with some things, such as learning about feeding and nappy changing, because he had prior experience of that. Overall, the narratives of these heavily engaged fathers showed an eagerness to learn, and that the relationship with the family nurse grew and deepened over time. Some men had strong views about what they did and did not need help with, and they and the nurses sometimes had differing views about what family members needed and what ‘full’ or ‘active’ engagement and participation in such a programme meant.

## Partially engaged fathers

For some fathers, while engagement with the FNP programme occurred, it was irregular and limited in nature. A range of dynamics caused this pattern of partial engagement. In some instances, low engagement was straightforwardly due to the fact that the family nurses visited during the day, when the father was absent at work or in training or education. A crucial structural reason for family nurses having ambiguous and sometimes tenuous relationships with fathers is that the FNP regards the mother as the primary client. Some men were very clear about the consequences of this:

They should involve the father more and want to see them all the time, just like they do the mothers. (17 yr old; child 6 months; White British)

Try to get us [fathers] more involved with activities basically. Like I mentioned earlier about it, like an evening time appointment so I’m able to receive information and be there when the family nurse is there and be able to hear information myself, not off [my partner] and ask any questions I want to ask. Sometimes I say ‘[partner's name], oh when's the family nurse coming?’ She says, ‘oh tomorrow’, and I say well can you ask her this. I’ve said that a few times. (24 yr old; child 6 months)

Partial engagement also happened with some fathers when they were present when the family nurse visited and most or sometimes all of the attention was on the mother (and baby). The findings suggest that the power of the nurse to determine who is involved, and how, in the sessions was highly significant. However, on some occasions it was not as simple as a well-motivated father being ignored by a mother-centred practitioner (good service user/bad professional dualism). A range of factors in combination produced partial engagement.

Mike's experience illustrates this well. He is Black British and was twenty-one when interviewed for the research and lived with his nineteen-year-old White British girlfriend, ‘Rose’ and their seven-month-old baby. They had been together for four years. Both partners were present during the research interview, when Mike's limited involvement with the FNP was raised:

Mike: We were referred by our midwife. It was about four months into the pregnancy. It is really for . . . I don't know – just to try and help us out I suppose. I am at work most of the times she comes – I am only there for about one in every four [family nurse visits] really. She has come in an evening but not much. So I don't see her much. I would like to be here more but it is sometimes nice to come home and relax after work and not spend an hour with a nurse. I do want to be here more often because I miss out on what she tells us. She always contacts [Rose] to arrange visits.

Rose: I never really said we needed [Mike] there. I didn't know I could do that. Because I thought it was all for mums really. When he has been here, he has just sat there.

Mike: Even when I am here, she [family nurse] doesn't really bring me in. She aims the questions at Rose not me. I feel like I am sat here and am nothing to do with it really. It is all about Rose and the baby. A lot of the sheets are about mother and baby. There is the odd sheet I have to fill out but the majority ain’t. She leaves me things to do but I don't always do them, some I think are a bit too personal.

Here we have a father who feels marginalised by the FNP because they often visit when he cannot be there and ignore him when he is. Mike claims to want to be involved, but would like it to happen at a time when he is not feeling tired. If the family nurse is not including him in the sessions when he is present, then responsibility for excluding him lies there. He does, however, make some contribution to his own exclusion (albeit less than the nurse, due to the power imbalances) in choosing not to engage when he is there and resisting the programme because it is too ‘personal’. His partner has contributed to the situation in apparently not knowing that the service is also for fathers, so she has not included him or challenged the family nurse to do so. While this reveals the influence of the mother being the primary FNP client, and the ultimate power of the professional and organisation to shape encounters, the partial engagement of the father was, in part, the outcome of several interacting factors and the dynamics between the professional, mother, father and baby.

A further pattern to emerge within these dynamics was that the greater the risk to the child, and the higher the support needs of the mother, the greater the risk of the family nurse engaging the father in partial ways. As one family nurse put it, ‘I’ve enough to do trying to help the mother’ (see also, Ofsted, 2011). One variation of this was where the father was viewed as an adequate parent but as a risk to the mother, and the FNP approach was to only partially involve him as a result. This was the case with Raymond, who was nineteen at the time of interview. His partner, Gloria, was twenty, and their child, Samantha, was five months old. Raymond was unemployed and they were very poor. He was very heavily involved during the pregnancy, and Gloria, who stayed in the room for the research interview, agreed that he shares the parenting and feels he is a ‘really good dad’. Both Raymond and Gloria initially felt quite positive about the involvement of the family nurse, but felt that her visits now mainly focus on mother and baby:

Raymond: I just want to be more involved with it. Though she includes me the most of anyone else, I’m not as involved as I should be. Most of the paperwork is down to Gloria – if it was half and half it would be okay, but it's like 80 per cent Gloria. Most of the sheets are for the mother. There's a few for the dad.

Gloria: It sometimes says this is for the dads but most of it is for the mum. I think she feels I understand more.

Raymond worries that the family nurse sees him as childish, and both he and Gloria have felt some animosity towards her since she got social workers involved on two occasions. The family nurse's position in the research interview was that she twice referred them to social care due to concerns about their complex needs that could develop into child neglect. She regards Raymond as having strengths as a father, but as immature and demanding on his partner, who is extremely vulnerable. The relative lack of attention she gives to the father is part of a strategy to provide as much support as possible to a highly vulnerable young mother, who was seriously abused by her own parents, and to a degree is still at risk of being harmed by them. The complexity then is that a man, who is regarded as a resource in providing adequate direct care for his child, is also seen as a risk to the child's well-being because of the demands he makes on the vulnerable mother. From the FNP's perspective, this approach appeals as a way of promoting the mother and child's safety, while the father feels it is unfair. And the risk remains because nothing is done to try to change him and his parenting.

Partial or non-engagement of fathers is particularly problematic when it means that opportunities are missed to develop men's caring capacities. Lee was eighteen at the time of interview and his partner, Jane, was seventeen. Both are White British and their son, Alfie, was fifteen months old. Lee spoke of how the pregnancy changed him and motivated him to be an involved father. He felt awkward at crying at the birth and struggled then, and still does to an extent, to know how to be in public as a man and father:

I think I was embarrassed really because I didn't really know anything and was learning all the stuff. I don't think she helped me get over being embarrassed, I can't remember. She was more focussed on [Jane]. She didn't involve me a lot. I guess I could involve myself more saying can I do this but I am actually embarrassed to butt in. If he [Alfie] was born now, I’d be a bit more pushy. I don't think I was bothered because I was young.

Lee takes some responsibility for not being more proactive in trying to get the family nurse to recognise him as a father and to present his need for support. But as a seventeen-year-old young man only recently out of school, he did not know how to ask for help, worried that doing so and showing his feelings compromised his identity within the traditional parameters of masculinity, and struggled to find a place in what he experienced as the feminine world of baby care. There was evidence that such confusion, ambivalence and passivity was sometimes perceived by the professional as disinterest and that this provided further justification for partial or non-engagement. The family nurses in this study had generally positive views of men and what fathers had to offer, but when faced with the complex demands of managing home visits and supporting high-need mothers, they sometimes misinterpreted men's embarrassment, insecurity and uncertainty as lack of interest. Ongoing critical attention to gender and masculinity through training and staff development which enables professionals to routinely examine their own assumptions, beliefs and experiences, is essential in such work (Fletcher and Visser, 2008). Early intervention and safeguarding work can support young fathers to develop and enact masculinities based on nurture and the legitimacy of showing and talking about feelings.

## Non-engaged and hostile fathers

The final engagement pattern to emerge involved fathers who are resistant towards, and sometimes hostile about, professional involvement. Resistant and hostile men were those who were actively non-engaged in the sense of seeming determined to avoid the service. One source of resistance in such cases is where the man's behaviour is controlling and abusive and he fears it will be exposed by professionals. ‘Dave’, who was interviewed in prison where he was serving a nine-year sentence for violence, and who was quoted earlier in the article, said he would be unwilling to cooperate with FNP involvement on his release. He had never engaged with the FNP as he went to prison before they became involved. The family nurse was concerned because his partner Elaine, with whom she had a close working relationship, spoke about feeling controlled by him from a distance through endless telephone calls. The family nurse was using motivational interviewing techniques to enable Elaine to come to an understanding of the kind of relationship she was in with Dave and to consider whether to continue it. In the interview, Dave made it clear that when he is released into the community he will have nothing to do with programmes like the FNP and nor will Elaine:

To be honest with you, if I was out I wouldn't want them there. I don't think [Elaine] would either. She only needs them because I’m not there.

The family nurse's perspective was the opposite to the father’s, regarding the danger he represented for Elaine and the baby as a key reason for their involvement.

An instructive example of a man who did have FNP involvement, and who absolutely hated it, is ‘James’, who was twenty when interviewed. His partner ‘Hannah’ was eighteen and their baby was four months old. The research interview took place in prison, where James had been for three months. He was convicted of drugs offences and had a further eight months to serve. James recalled the family nurse:

Father: I think I’ve met her about twice. She wrecks my head. She's just too nosy. She just asks questions that aren't relevant really. Things that have nothing to do with her. I used to just go out or I’d be at probation. I just don't like her and I’m not bothered if she knows I don't like her. I don't even get why she comes round . . . She tried to give me leaflets and something to fill out, but I don't know if I done it. She gave them to me and told me what I had to do with them and that's it. I didn't fill them in. She's just a head wrecker. She's like she's part of the relationship. She wants to know too much.

Interviewer: Is there nothing you feel you have needed help with as a dad?

Father: Nope! . . . I don't like her. She just annoys me, the shit she asks about. She may think it's relevant, I don’t.

To understand the possible sources of such resistance and hostility it is necessary to place it in the context of James’ biography. He and Hannah had been going out for just a few weeks when she got pregnant. ‘At that time I was smoking it [cannabis] all the time.’ He had been ‘kicked out’ of home by his mother and was on a community order for a burglary offence and had an electronic tag, but was allowed to stay at Hannah's for the birth. The pregnancy was a ‘shock’.

I’m not one of those who would just fuck off. Most of my time I’ve grown up without my dad and I know how horrible it is to grow up like that. I knew I wanted to stick by her and that. I didn't want my daughter to grow up without a dad. Just because I have doesn't mean she has to.

James’ father has been in prison many times for theft, ‘he's a twat’. Having his daughter ‘was one of the best feelings ever’. He had five weeks at home with the baby before going to prison and says he was active in caring for her. This is his first time in prison and ‘it's pretty shit’. Hannah and the baby visit him once a week and fatherhood has changed him for the better: ‘I changed before I came in, thinking about my daughter and my missus . . . I need to look after her and I can't do it in here.’

He has not received any FNP contact in prison, and fully expects to be a father in the community without their assistance. He took every opportunity to be critical of the family nurse, especially her desire to be ‘part of the relationship’.

The family nurse should do things that are relevant instead of being part of the relationship. She just wants to be too involved and that. She needs to chill out a bit. I think the family nurse's job is to be there for the baby and mother but she's not, she's just trying to be part of the relationship. What they do should be about weighing the baby and that. I don't think she has any right asking [partner] what she does with money. She can't help me with the things I’ve got to do. If she's gonna be there, be there for the baby's sake, not the relationship's sake.

Such resistance and anger could be interpreted as a reasonable response to a model of relationship-based service delivery that the service user does not want. But once again, we need to go further than an interpretative framework based just on rights to a more dynamic psycho-social one that takes account of gender, class, the psyche and emotional life. James’ resistance and hostility appears to be rooted in the ‘social suffering’ (Frost and Hoggett, 2008) which arises from a combination of current and past experiences of poverty, adversity, traumatic intra-familial relationships and oppressive encounters with the law and health and social care professionals. James’ oppressive experiences of the State have led him to internalise the belief that all professionals are to be mistrusted and kept at a distance, so much so that his opposition to the family nurse and any kind of intervention into his life is visceral. Professional interest in him, and even attempts at care and empathy are now experienced as invasion, intimidation and control. This drive to defend the self is compounded by acute disappointments in family relationships, in particular his loss from his father being a recidivist prisoner, and his sadness and anger with himself for going to prison and beginning to visit on his own child what his father did to him.

## Conclusion

This article has identified three broad patterns of engagement and non-engagement of vulnerable young fathers in the early intervention and safeguarding work of the Family Nurse Partnership and within these a range of factors and dynamics that influenced practice and the men's and their family's experiences. Some of the men who engaged positively with the FNP programme did so from a starting-point of suspicion with a risk of non-engagement. The fact that early intervention managed to successfully engage some initially suspicious fathers provides a basis for learning and developing possible strategies to engage fathers who found the FNP service hard to use and who for a variety of reasons are wary of seeking or receiving advice from services. Fathers’ vulnerabilities, especially in the face of powerful professionals, and their reasons for refusing services, need to be taken into account, and the article has identified some areas in need of change. It cannot be known at the outset which fathers will be reachable and engage and who will not. It was through adopting an approach of creative persistence that family nurses managed to fully engage some reluctant fathers, some of whom spoke of how they were won round: ‘Family nurses could tell the fathers they are needed at the meetings, like when [family nurse] encouraged me to stay in the house.’ The family nurses had a repertoire of skills and tactics that they used to encourage men to become involved and developed meaningful relationships within which they managed to appeal to the men's interest in caring for their babies and their desire to have the best for their child (Ferguson and Gates, 2015).

Further research is needed into men who find it hard to use services. But from the glimpses gained of them in this article, the hostility of men who will not engage arises partly from the nature of the service and the ways it is delivered, and also seems to come from their deep experience of social suffering and pained defensiveness that makes isolation and being unreachable highly attractive. But, as a strategy for developing into a good enough father, it is highly unlikely to work and is self-defeating. Getting close to such men and penetrating their defences, represents a huge professional challenge. For the thing they want least is precisely that which this research has shown can help young fathers: a sustained therapeutic and supportive relationship within which they can learn from experiencing the kind of consistency, nurturing, discipline and skilled care they need to pass on to their babies.
